# Differential DNA extraction of challenging simulated sexual-assault samples: a Swiss collaborative study

**DOI:** 10.1186/2041-2223-2-11

**Published:** 2011-05-04

**Authors:** Séverine Vuichard, Urs Borer, Michel Bottinelli, Christian Cossu, Naseem Malik, Verena Meier, Christian Gehrig, Andrea Sulzer, Marie-Laure Morerod, Vincent Castella

**Affiliations:** 1 Unité de génétique forensique, Centre universitaire romand de médecine légale, Centre Hospitalier Universitaire Vaudois et Université de Lausanne, rue du Bugnon 21, 1011 Lausanne, Switzerland; 2 Institut für Rechtsmedizin, Abt. Forensische Molekularbiologie, Sulgenauweg 40, 3007 Bern, Switzerland; 3 Laboratorio di diagnostica molecolare, c/o Cardiocentro Ticino, Via Tesserete 48, 6900 Lugano, Switzerland; 4 Institute of Legal Medicine St. Gallen, Forensische Genetik, Rorschacherstrasse 93, 9007 St. Gallen, Switzerland; 5 Institut für Rechtsmedizin, Pestalozzistrasse 22, 4051 Basel, Switzerland; 6 Unité de génétique forensique, Centre Universitaire Romand de Médecine Légale, Avenue de Champel 9, 1206 Genève, Switzerland; 7 Institute of Legal Medicine, University of Zurich, Winterthurerstrasse 190, 8057 Zurich, Switzerland

## Abstract

In sexual-assault cases, autosomal DNA analysis of gynecological swabs is a challenge, as the presence of a large quantity of female material may prevent detection of the male DNA. A solution to this problem is differential DNA extraction, but there is no established best practice for this. We decided to test the efficacy of a number of different protocols on simulated casework samples. Four difficult samples were sent to the nine Swiss laboratories active in forensic genetics. In each laboratory, staff used their routine protocols to separate the epithelial-cell fraction, enriched with the non-sperm DNA, from the sperm fraction. DNA extracts were then sent to the organizing laboratory for analysis. Estimates of male:female DNA ratio without differential DNA extraction ranged from 1:38 to 1:339, depending on the semen used to prepare the samples. After differential DNA extraction, most of the ratios ranged from 1:12 to 9:1, allowing detection of the male DNA. Compared with direct DNA extraction, cell separation resulted in losses of 94-98% of the male DNA. As expected, more male DNA was generally present in the sperm than in the epithelial-cell fraction. However, for about 30% of the samples, the reverse trend was seen. The recovery of male and female DNA was highly variable, depending on the laboratory involved. An experimental design similar to the one used in this study may be of assistance for local protocol testing and improvement.

## Background

When analyzing samples from sexual-assault cases, such as gynecological swabs, forensic-genetics laboratories aim to establish the autosomal DNA profile of the male contributor to help identify its source. The success of these analyses depends upon several factors: the circumstances of the case (number of aggressors, presence/absence of ejaculation, etc.), the aggressor's semen characteristics, time elapsed between the aggression and the collection of gynecological swabs [1-3], and the sampling and storage conditions. Another important factor, which is seldom assessed, is the analytical process itself. Indeed, experimental studies have shown that protocol variation can influence the success of DNA analysis [4-6].

Samples from sexual-assault cases are often characterized by imbalanced mixtures of epithelial cells and sperm, with an excess of the victim's material, resulting in an unfavorable ratio of male to female DNA. According to several studies [7,8] and our own internal validations, the male autosomal DNA component of the mixture is too low to be detected beyond a ratio of 1:10 to 1:20 of male:female DNA. This is essentially due to competition for the primers during PCR amplification, which leads to preferential amplification of the major component of the mixture. In such cases, the use of Y-chromosome genetic markers, such as short tandem repeats (STRs), may allow the amplification of low quantities of male DNA independently of the victim's DNA background [7-10]. However, a Y-STR profile is not as informative as an autosomal STR profile. First, paternally related males cannot be discriminated. Second, the frequency of a Y-STR profile in the population can be relatively high [11], impeding the discrimination of some unrelated males. Third, Y-STR profiles are often not included in national DNA databases. Therefore, the acquired Y-STR profile can generally only be compared with the Y-STR profile of known suspects.

Hence, forensic-genetics laboratories try to separate the male from the female material to increase the chances of obtaining the perpetrator's autosomal profile. Several cell-separation techniques exist: differential lysis, which relies on the differential resistance of spermatozoa and epithelial cells to chemicals [12,13]; laser microdissection, which allows the excision and collection of spermatozoa, independent of the surrounding cellular material [14-16]; membrane filtration and microdevices that exploit differences between size and shape of the cells [17,18]; and flow cytometry, which takes advantage of specific membrane proteins to mark and sort cells [19]. Most forensic laboratories use differential DNA extraction, which does not require expensive equipment and is quickly achieved. Briefly, this technique includes a mild cell lysis step that allows the recovery of an epithelial-cell fraction enriched with DNA from the female's epithelial cells and leukocytes. A stronger cell lysis is then used to break the spermatozoa membrane and recover their DNA in the sperm fraction [12].

The aim of this preliminary study was to assess the effect of variation of the differential DNA-extraction protocols on the analysis success. For this purpose, samples consisting of a mixture of epithelial cells from buccal swabs and dilutions of semen were sent to the nine Swiss laboratories active in forensic genetics. They were asked to perform DNA extraction as they would in real cases, and to send back all DNA extracts. We then measured the amounts of male and female DNA recovered from the epithelial-cell and sperm fractions using real-time quantitative PCR (qPCR). DNA extracts were further amplified with autosomal DNA STRs and Y-STRs. To evaluate the DNA yield of differential DNA extraction, samples containing either semen dilutions or epithelial cells were processed in our laboratory without differential lysis. The obtained results are presented and discussed below.

## Results

The nine participating laboratories used differential-extraction protocols to analyze the simulated sexual-assault samples. The chemicals used for cell lysis, the number of washing of the spermatozoa pellet, and the way in which the DNA was purified and concentrated differed between laboratories (Table 1). However, it should be noted that the DNA quantification and DNA profiling were standardized because they were all performed in the organizing laboratory.

**Table 1:** Differential DNA extraction protocols

Laboratories	First cell lysis	No. of washes	Second cell lysis	DNA Purification	Concentration devices (laboratory number)
1, 3	Differex^a^; proteinase K	2	ATL^b ^buffer^c^; DTT^d^; proteinase K	QIAamp DNA mini kit^c^	Microcon^e^
2, 4, 7, 9	Lysis buffer^f^; proteinase K	1, 3, 4, 3	Lysis buffer^f^; DTT^d^; proteinase K	Organic	Precipitation (2,4,7); Centricon^e ^(9)
5	Chelex^g^; proteinase K	3	Chelex^g^; DTT^d^; proteinase K	Chelex^g^	Centricon^e^
6	Lysis buffer^f^; proteinase K	3	DTT^d^; proteinase K	QIAamp DNA mini kit^c^	Centricon^e^
8	ATL^b ^buffer^c^; proteinase K	3	ATL^b ^buffer^c^; DTT^d^; proteinase K	QIAamp DNA micro kit^c^	-

^a^Promega AG, Dübendorf, Switzerland.

^b^Animal Tissue Lysis.

^c^Qiagen AG, Hombrechtikon, Switzerland.

^d^Dithiothreitol.

^e^Millipore AG, Zug, Switzerland.

^f^Home-made solution.

^g^Bio-Rad Laboratories AG, Reinach BL, Switzerland.

### Male and female DNA separation

We used qPCR to assess the quantities of male and female DNA recovered in the sperm and epithelial-cell fractions after differential DNA extraction. All laboratories succeeded in enriching the sperm fraction with male DNA. In particular, for samples prepared with semen from volunteer 1, the median values of male and female DNA recovered in this fraction were 10.3 and 4.7 ng, respectively (Table 2). Therefore, the differential-extraction protocols allowed the recovery of about twice as much male as female DNA in the sperm fractions, or a 2:1 ratio. The opposite situation occurred in the epithelial-cell fractions that contained 3.3 and 3615.1 ng of male and female DNA, respectively (Table 2), corresponding to a 1:1095 ratio. When samples were prepared with semen from volunteer 2, the male DNA tended to move from the sperm to the epithelial-cell fraction. The sperm fractions contained 0.3 and 1.2 ng of male and female DNA, respectively (1:4 ratio), whereas the epithelial-cell fractions contained 0.8 and 2584.0 ng, respectively (1:3230 ratio) (Table 3).

**Table 2:** Data for swabs containing mixtures of epithelial cells and semen from volunteer 1 (data are mean ± SD unless otherwise stated).

Laboratory	Male DNA in SF, ng^a^	Female DNA in SF, ng^a^	Male:female ratio	Extract volume of SF, μl	Male DNA in EF, ng^a^	Female DNA in EF, ng^a^	Recovered male DNA in SF, %^b^	SGM Plus^c ^profiles (SF), ng^d^	Y loci, n^g^
1	26.5 ± 7.3	6.0 ± 17.3	5:1	**25**	**3.3 **± 1.1	**3615.1 **± 78.6	14	M (major) + F	11 (SF)
2	3.3 ± 2.2	**4.7 **± 7.4	1:1	40	7.4 ± 0.5	6729.6 ± 2628.1	2	M (major) + F	11 (EF)
3	18.1 ± 0.9	8.4 ± 16.3	**2:1**	**25**	4.7 ± 0.5	5367.0 ± 2352.6	10	M (major) + F	11 (SF)
4	13.0 ± 10.9	5.8 ± 27.6	**2:1**	22	4.3 ± 0.1	3801.7 ± 545.7	7	M	11 (SF)
5	ND^e^	1.5 ± 1.1	0	50	0.4 ± 0.1	0.1 ± 0.2	0	Not interpretable	8 (EF)
6	0.4 ± 0.2	2.1 ± 0.6	1:6	60	0.2 ± 0.2	45.1 ± 28.9	0	Not interpretable	11 (SF)
7	0.8 ± 0.2	ND	Indet.^f^	**25**	0.1 ± 0.2	50.0 ± 13.4	0	Not interpretable	11 (SF)
8	11.4 ± 5.8	9.7 ± 13.7	1:1	**25**	ND	387.8 ± 494.9	**6**	M (major) + F	11 (SF)
9	**10.3 **± 2.0	1.2 ± 4.0	9:1	50	3.4 ± 1.8	5765.2 ± 192.1	**6**	M	11 (SF)

Relevant median values are in bold.

^a^Mean quantities and percentages of male and female DNA recovered in the sperm (SF) and epithelial-cell fractions (EF) after differential DNA extraction are reported (means are from duplicate analyses of two different samples, that is, four values).

^b^100% correspond to the amount of male DNA recovered after direct DNA extraction of samples containing only semen, without epithelial cells

^c^Second Generation Multiplex Plus^® ^(Applied Biosystems).

^d^Characteristics of the SGM Plus DNA profiles (M = male DNA profile; M+F = mixed DNA profile; major = major component of the mixed DNA profile) and number of loci obtained with the PowerPlex Y kit are given.

^e^ND = none detected

^f^Indet. = indeterminate.

^g^PowerPlex Y System (Promega)

**Table 3:** Data for swabs containing mixtures of epithelial cells and semen from volunteer 2 (data are mean ± SD unless otherwise stated).

Laboratory	Male DNA in SF, ng^a^	Female DNA in SF, ng^a^	Male:female ratio	Extract volume of SF, μl	Male DN in EF, ng^a^	Female DNA in EF, ng^a^	Recovered male DNA in SF, %^b^	SGM Plus^c ^profiles (SF), ng^d^	Y loci, n^f^
1	2.2 ± 1.2	5.6 ± 6.1	**1:3**	25	**0.8 **± 0.1	**2584.0 **± 790.1	10	M + F (major)	11 (SF)
2	0.1 ± 0.1	1.5 ± 0.3	1:12	40	3.6 ± 1.7	3408.9 ± 1457.6	**1**	Not interpretable	11 (EF)
3	3.5 ± 0.2	1.7 ± 0.3	2:1	25	2.0 ± 0.3	2875.7 ± 602.6	16	M (major) + F	11 (SF)
4	0.8 ± 0.7	0.6 ± 2.4	1:1	22	1.6 ± 0.4	3836.6 ± 1454.3	4	Not interpretable	11 (EF)
5	ND^e^	**1.2 **± 0.7	0	50	ND	0.1 ± 0.0	0	Not interpretable	0 (SF/EF)
6	0.2 ± 0.0	0.8 ± 1.1	1:3	60	0.2 ± 0.2	500.2 ± 333.7	**1**	Not interpretable	7 (SF/EF)
7	ND^e^	0.4 ± 0.0	0	25	ND	67.3 ± 17.3	0	Not interpretable	7 (SF/EF)
8	1.7 ± 0.1	1.9 ± 0.5	1:1	25	ND	248.3 ± 22.9	8	Not interpretable	11 (SF)
9	**0.3 **± 0.4	0.2 ± 0.9	2:1	50	1.3 ± 0.4	5713.0 ± 394.6	**1**	Not interpretable	8 (EF)

Relevant median values are in bold.

^a^Mean quantities and percentages of male and female DNA recovered in the sperm (SF) and epithelial-cell fractions (EF) after differential DNA extraction are reported (means are from duplicate analyses of two different samples, that is, four values).

^b^100% correspond to the amount of male DNA recovered after direct DNA extraction of samples containing only semen, without epithelial cells

^c^Second Generation Multiplex Plus^® ^(Applied Biosystems).

^d^Characteristics of the SGM Plus DNA profiles (M = male DNA profile; M+F = mixed DNA profile; major = major component of the mixed DNA profile) and number of loci obtained with the PowerPlex Y kit are given.

^e^ND = none detected

^f^PowerPlex Y System (Promega)

The recovery of male and female DNA differed depending on the laboratories. For samples prepared with semen from volunteer 1, the amount of male DNA recovered in the sperm fraction varied between 0.0 and 26.5 ng (Table 2) and the amount of female DNA in the epithelial-cell fraction ranged from 0.1 to 6729.6 ng (Table 2). About the same level of variation was seen with the semen from volunteer 2 (Table 3). Laboratories 5, 6 and 7 used different DNA-extraction protocols (Table 1), but recovered the least male DNA (Tables 2 and 3). They were also those who recovered the least female DNA (Tables 2 and 3).

The results obtained with differential DNA extraction were compared with those obtained with direct DNA extraction. For this, DNA was directly extracted, without differential lysis, from six duplicated samples prepared exclusively with semen (diluted 1 in 50) or epithelial cells. Mean DNA concentrations of 7.55 and 0.85 ng/μl were obtained for the semen dilutions from volunteers 1 and 2, respectively. Consequently, the amount of DNA recovered from the swabs with direct DNA extraction was, respectively, 188.8 and 21.3 ng for the two donors (DNA-extract volume was 25 μl). The amount of male DNA recovered in the sperm fractions after differential DNA extraction represented only about 6% and 1% of those obtained for the two semen donors, respectively, with direct DNA extraction (Tables 2 and 3). A mean DNA concentration of 287.82 ng/μl was obtained for the six female volunteer buccal swabs, which corresponds to a quantity of 7195.5- ng DNA in volume of 25 μl of DNA extract. The loss due to the differential extraction was about 50 to 64% compared with direct DNA extraction from buccal swabs of the female donor. From the values above we would expect that, without differential DNA extraction, male:female DNA ratios of 1:38 and 1:339 would have been obtained for the simulated sexual-assault samples prepared with the semen from the two volunteers, respectively.

### DNA profiling

DNA profiling was used to determine whether DNA characteristics of the male contributor could be retrieved. On eight out of 18 occasions, laboratories obtained male autosomal DNA profiles, either pure or mixed with the female DNA profile, for samples prepared with semen from the two donors (laboratories 1, 2, 3, 4, 8 and 9 (Table 2) and laboratories 1 and 3 (Table 3)). Male DNA concentrations (Tables 2 and 3, columns 2 and 5) and male:female DNA ratios (Tables 2 and 3, columns 2 and 3) of the corresponding sperm fractions ranged from 0.09 ng/μl (laboratory 1; Table 3) to 1.06 ng/μl (laboratory 1; Table 2), and from 1:3 (laboratory 1; Table 3) to 9:1 (laboratory 9; Table 2), respectively. The ratios generally allowed prediction of whether the female or the male was the major or minor component of the mixed DNA profile.

DNA profiles obtained with the sperm fractions in the other laboratories could not be interpreted; they were not reproducible between the two amplifications because of the occurrence of false alleles (drop-in) and allele loss (drop-out). The samples concerned were characterized by male DNA concentrations ranging from 0.00 ng/μl (laboratory 5 (Tables 2 and 3) and laboratory 7 (Table 3)) to 0.07 ng/μl (laboratory 8; Table 3), and male:female DNA ratios ranging from 0 (laboratory 5 (Tables 2 and 3) and laboratory 7 (Table 3)) to 2:1 (laboratory 9; Table 3) and 1:0 (laboratory 7; Table 1), respectively.

Apart from one exception (laboratory 5; Table 3), full (11 out of 11 loci scored) or partial (<11 loci scored) Y DNA profiles were obtained for both semen qualities within each laboratory (Tables 2 and 3). The fractions containing the highest amount of male DNA were amplified. These were generally the sperm fractions for samples prepared with semen from volunteer 1, except for the samples analyzed at laboratories 2 and 5 (Table 2). When samples were prepared with semen from volunteer 2, the highest amount of male DNA was found three times in both the sperm (laboratory 1, 3 and 8) and the epithelial cell (laboratory 2, 4 and 9; Table 3) fractions. Both fractions were reported by the three remaining laboratories to contain comparable amounts of male DNA (Table 3).

## Discussion

This study was undertaken to assess the effect of various differential DNA-extraction protocols on the success of analysis of challenging simulated sexual-assault samples.

Differential DNA extraction was first described in 1985 [12]. Compared with other methods, such as flow cytometry [19] and laser microdissection [14-16], it is relatively quick and low-cost. The simulated sexual-assault samples prepared for the present study contained a large excess of female material. Without differential DNA extraction, estimates of male:female DNA ratios were 1:38 and 1:339 for samples prepared with semen from volunteers 1 and 2, respectively.

It is generally accepted that the autosomal DNA characteristics of the minor contributor of a DNA mixture cannot be detected when ratios exceed 1:10 to 1:20 [7,8]. Therefore, male autosomal DNA profiles extracted from the simulated sexual-assault samples would not have been detected without cell separation. After differential DNA extraction, laboratories that succeeded in detecting the autosomal DNA characteristics of the male contributor had male:female DNA ratios ranging from 1:3 to 9:1, and male DNA concentrations ranging from 0.09 to 1.06 ng/μl. Compared with a direct DNA-extraction protocol, differential DNA extraction generates losses of about 50 to 64% of female DNA in the epithelial-cell fractions, and about 94 to 98% of male DNA in the sperm fraction. Differential DNA extraction therefore requires a relatively high level of male material to be present in the original sample. It would have been interesting to compare these yields with those from other cell-separation techniques; unfortunately, comparable data are presently not suitable for such comparisons.

Unexpectedly, there was marked variation in the success of analysis between laboratories. In the analysis of identical simulated sexual-assault samples, some laboratories obtained pure autosomal male DNA profiles, whereas others did not. Sample preparation and DNA analyses would have influenced this observation only marginally; indeed, the study was designed to minimize their effect. Samples were randomized twice (after buccal swab collection, and after semen deposit), so that the order in which they were prepared would not favor any of the laboratories. In addition, the performance of the laboratories in recovering DNA from the samples that were prepared independently with semen from volunteers 1 and 2 was similar, indicating that sample preparation had a negligible effect compared with the variation produced by the different protocols for differential DNA extraction. In some cases, there was a shift of male DNA from the sperm to the epithelial-cell fractions for samples prepared with semen from both volunteers, confirming that cell separation plays a major role in the process. Lastly, DNA quantification and DNA profiling were all performed in the organizing laboratory; they were therefore standardized and did not influence the results.

Six of the nine participating laboratories obtained male autosomal DNA profiles, either pure or mixed with the female DNA profile, for samples prepared with the semen from volunteer 1. By contrast, only two of the nine laboratories obtained the autosomal DNA short tandem repeat (STR) characteristics of the male contributor when samples were prepared with the semen from volunteer 2. Both successful laboratories used the same system (Differex; Promega AG, Dübendorf, Switzerland), which seems to offer a good compromise between male DNA loss and male DNA purity. Similar results have been obtained in two previous studies [6,20].

Losses of male DNA resulting in unfavorable male:female DNA ratios (from 1:12 to 2:1) and/or in DNA extracts that were too dilute (from 0.00 to 0.07 ng/μl) resulted in DNA profiles that were not interpretable in the other laboratories. Corresponding electropherograms generally identified DNA mixture characteristics, but results were not reproducible between duplicate amplifications, because of the occurrence of false alleles and drop-out associated with the analysis of minute amounts of DNA [21].

Differential DNA-extraction protocols contain many parameters. This preliminary study was not sufficient to evaluate the effects of individual parameters within the nine participating laboratories. The initial lysis is likely to have a major influence on male DNA recovery. On one hand, an initial lysis that is too strong can break not only the epithelial-cell membranes, but also some of the spermatozoa, leading to the displacement of a significant amount of male DNA into the epithelial-cell fraction. On the other hand, an initial lysis that is too weak may not break all the epithelial-cell membranes leading to a contamination of epithelial cells in the sperm fraction. Other steps, such as cell elution from the swabs, also influence DNA recovery [5,22]. Incomplete elution might explain why, in our study, the laboratories who recovered the least male DNA were also those who got the least female DNA.

There may be several reasons why a larger proportion of male DNA from the semen of volunteer 2 was present in the epithelial-cell fraction compared with semen from volunteer 1. The semen from volunteer 1 contained almost nine times the level of DNA as that from volunteer 2. Furthermore, the semen from volunteer 1 was fresh, whereas the semen from volunteer 2 had been stored for 2 years at -20°C before the experiment, and might have been weakened. Lastly, semen characteristics, particularly spermatozoa robustness, may vary between individuals.

Unlike autosomal DNA STR profiles, Y-STR profiles were obtained for all samples except one. Autosomal DNA STRs were amplified with 28 PCR cycles, whereas 34 PCR cycles were used for Y-STRs, partly explaining the difference. Another important factor was that single-source Y-STR profiles were generally robust across duplicate amplifications, whereas variation between the allelic content of mixed autosomal DNA profiles impeded the interpretation of the results.

Interestingly, one-third of Y-STR profiles were obtained from the epithelial-cell fraction rather than from the sperm fraction. This has important consequences for real sexual-assault cases. A significant proportion of male DNA can be displaced into the epithelial-cell fraction, especially when the spermatozoa are weakened. This typically becomes more likely as the time elapsed between the rape and the collection of the gynecological samples increases. In such situations, the results show that the analysis of the epithelial-cell fraction may increase the chance of establishing the Y-STR profile of the male contributor.

Another important observation was that some Y-STR profiles were obtained even though no male DNA had been detected by the qPCR assay. Similar observations were previously made for autosomal markers [23], and these results suggest that current qPCR quantification methods are not sufficiently robust to suggest that no DNA occurs in a sample with a quantification close or equal to zero. Nevertheless, such data are helpful to select which samples, fractions (sperm or epithelial-cell) and DNA markers (autosomal or Y) should be analyzed to maximize the chance to help identify the person carrying out the assault.

## Conclusions

Ideally, the chance to establish a male DNA profile in a vaginal swab should not depend on the laboratory where the sexual-assault samples are analyzed. Nonetheless, each laboratory works with its own validated protocols, whose efficiency may fluctuate. National and international proficiency-testing programs, such as the German DNA Profiling Group (GEDNAP) [24], contribute to the consistency across laboratories. However, challenging samples, such as those analyzed in the present study, are seldom considered in these programs. Our preliminary study shows important differences between the differential DNA-extraction protocols tested, and the need for local optimization. An experimental design similar to the one used in this study may help laboratories improve their protocols by isolating the step, manipulation, chemical, instrument, consumable or conditions they want to test.

Another important finding from this study is that >90% of the male DNA initially present in the simulated sexual-assault samples was lost after differential DNA extraction. This is certainly problematic when analyzing gynecological samples with little male DNA present, such as those collected several hours or days after the incident. This enhances the need for the development and comparative testing, of alternative cell-separation techniques. Complementarily, methods such as microarray technology, high throughput DNA sequencing or analysis of SNPs panels [25], which allow access to the minor component of unbalanced DNA mixtures, may in the future help improve the success rate of DNA mixture analysis.

## Methods

### Sample preparation

Simulated sexual-assault samples were prepared by depositing 10 μl of semen diluted 1:50 in sterile water onto buccal swabs from one female volunteer. Preliminary tests found that obtaining a male autosomal DNA STR profile for samples prepared with this material is challenging when using a differential lysis protocol. The 1:50 semen dilution further allowed transfer by pipette of an apparently homogenous solution, without visible aggregates.

Buccal swabs were prepared by rubbing the inside of the cheek vigorously back and forth 10 times, leaving at least 2 hours between two successive samplings to minimize inter-sample variation.

Swabs were randomized, dried and stored at room temperature until semen deposition. Fresh semen from volunteer 1 was then deposited on samples A and B, and frozen semen about 2 years old from volunteer 2, was deposited on samples C and D. Samples were again randomized, dried for 2 hours at room temperature after semen deposition, and then sent to the nine participating laboratories.

Extra samples were prepared to evaluate the efficiency of differential compared with direct DNA extractions. They comprised 12 swabs containing 10 μl of semen from the two donors (6 swabs each) diluted 1:50, without epithelial cells, and six buccal swabs from the female volunteer, without semen. These samples were treated in the same way as those described above, except that they were extracted with a direct extraction technique (QIAamp/QIAshredder; Qiagen AG, Hombrechtikon, Switzerland) [26] in our laboratory without cell separation.

### Sample analysis

Various differential DNA-extraction protocols were used to separate the male and female DNA contained in the simulated sexual-assault samples (Table 1). All DNA extracts were sent to our laboratory and frozen upon receipt. They were quantified twice each with the two different kits (Quantifiler Human DNA Kit and Human Male Y DNA Quantification Kit; Applied Biosystems, Zug, Switzerland) using a qPCR analyzer (ABI 7300; Applied Biosystems) according to the manufacturer's instructions, except we used a final reaction volume of 12.5 μl. Because the DNA-extract volumes differed between the nine laboratories, we worked with total DNA quantities, rather than DNA concentrations. Means and standard deviations were calculated between duplicated samples. Female DNA quantities were obtained by subtracting male DNA from total DNA quantities.

Autosomal DNA and Y-STR profiles were established to evaluate whether the different protocols allowed the isolation of the male contributor. Autosomal DNA STR profiles were established with a commercial kit (SGM Plus Kit; Applied Biosystems) following the manufacturer's instructions, but with a 12.5 μl final reaction volume. For each sample and laboratory, the fraction of the DNA extract containing the highest concentration of male DNA was amplified using a Y-STR multiplex kit (PowerPlex Y System; Promega) according to the manufacturer's instructions, but using 34 PCR cycles. Thermal cyclers (GeneAmp 9700; Applied Biosystems) were used to amplify extracted DNA. Amplicons were separated using a genetic analyzer (ABI 3100; Applied Biosystems) and analyzed with the appropriate software (GeneMapper *ID*, version 3.2; Applied Biosystems) using standard procedures. Each DNA profile was confirmed with a second amplification. For the interpretation, all peaks with a height in excess of 50 relative fluorescence units on the electropherogram were considered, and were scored as alleles when they appeared in both amplifications. The DNA profiles were considered as uninterpretable when their allelic content was not reproducible between the two amplifications.

## Competing interests

The authors declare that they have no competing interests.

## Authors' contributions

MLM and VC conceived of the experiments. MLM prepared the samples and performed the quantitative real-time PCR and STR amplifications. SV and VC analyzed the data and drafted the manuscript. UB, MB, CC, NM, VM, CG and AS were involved in DNA extraction and manuscript improvement. All authors have read and approved the final manuscript.
